# The Multifaceted Roles of Fungal Cutinases during Infection

**DOI:** 10.3390/jof8020199

**Published:** 2022-02-18

**Authors:** Gulab Chand Arya, Hagai Cohen

**Affiliations:** Department of Vegetable and Field Crops, Institute of Plant Sciences, Agricultural Research Organization (ARO), Volcani Center, Rishon Lezion 7505101, Israel

**Keywords:** plant cuticle, cutin polymer, cutinase, pathogenic fungi, plant–pathogen interactions

## Abstract

Cuticles cover the aerial epidermis cells of terrestrial plants and thus represent the first line of defence against invading pathogens, which must overcome this hydrophobic barrier to colonise the inner cells of the host plant. The cuticle is largely built from the cutin polymer, which consists of C_16_ and C_18_ fatty acids attached to a glycerol backbone that are further modified with terminal and mid-chain hydroxyl, epoxy, and carboxy groups, all cross-linked by ester bonds. To breach the cuticle barrier, pathogenic fungal species employ cutinases—extracellular secreted enzymes with the capacity to hydrolyse the ester linkages between cutin monomers. Herein, we explore the multifaceted roles that fungal cutinases play during the major four stages of infection: (i) spore landing and adhesion to the host plant cuticle; (ii) spore germination on the host plant cuticle; (iii) spore germ tube elongation and the formation of penetrating structures; and (iv) penetration of the host plant cuticle and inner tissue colonisation. Using previous evidence from the literature and a comprehensive molecular phylogenetic tree of cutinases, we discuss the notion whether the lifestyle of a given fungal species can predict the activity nature of its cutinases.

## 1. Introduction

The terrestrialisation of plants is considered to be a landmark event in plant evolution [1,2]. Key to this major step was the assembly of the cuticle, a lipophilic barrier that covers all plant aerial tissues, including the leaves, stems, flowers, and fruit [3]. The cuticle is made primarily from the polyester cutin, which consists of C_16_ and C_18_ fatty acids attached to a glycerol backbone that are further modified with terminal and mid-chain hydroxyl, epoxy, and carboxy groups, all cross-linked by ester bonds [4]. The cutin matrix is immersed in and coated with solvent-extractable cuticular waxes composed of various C_20_-C_40_ very-long-chain fatty acids (VLCFAs) as well as their esters and derivatives [5]. Besides fulfilling pivotal roles in the regulation of transpiration and the diffusion of gases, water, and solutes [6,7,8], the cuticle is also the first line of defence against a wide range of pathogens as it covers the outermost epidermal layer of aerial organs. Pathogens must penetrate the cuticle to colonise the nutritional inner cells of the host plant: some gain entry through stomatal pores, natural cracks, or wounds, whereas many others utilise an arsenal of strategies to breach the cuticle either by using mechanical pressure to pierce its surface or by degrading cutin using specialised hydrolytic enzymes known as cutinases. Although the cuticle covers all above ground organs, the tissues that built the outer bark of trees is made of a hetero-polymer called suberin, which is more complex than cutin as it is built from longer-chain fatty acids and aromatic *p*-hydroxycinnamic acids derived from the core phenylpropanoid pathway. It is assumed that pathogenic fungal species that are capable of penetrating these types of tissues possess unique enzymes called suberinases that are capable of degrading the suberin polymer. Nonetheless, research on suberinases is scarce with a very limited knowledge regarding the mechanisms allowing these enzymes to degrade suberin.

**Figure 1:**
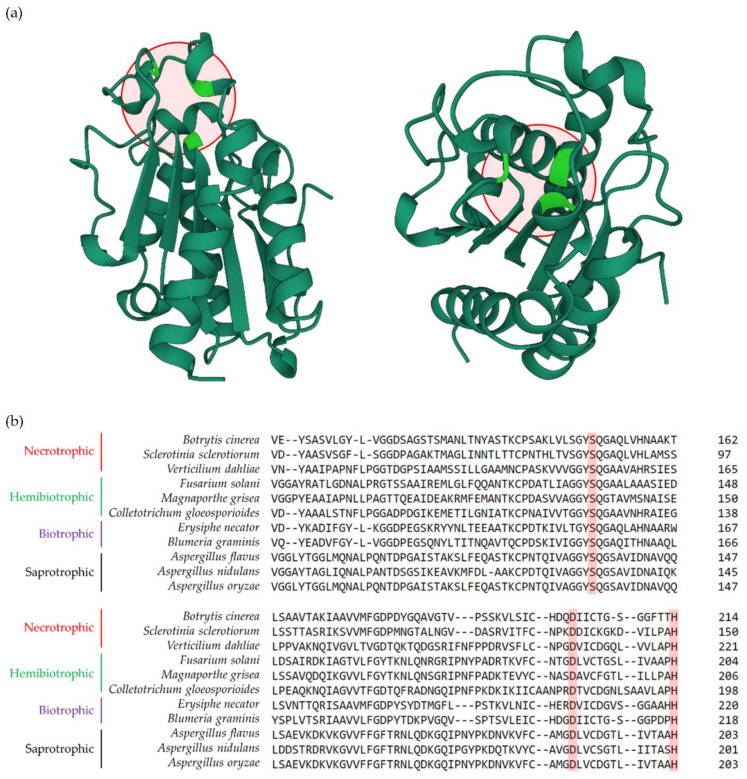
Structure of the *Fusarium solani* cutinase and the conserved catalytic triad among fungal species with different lifestyles. (**a**) The crystal structure of the *Fusarium solani* CUT enzyme (protein ID: 1CEX) according to the RCSB Protein Data Bank (PDB) (https://www.rcsb.org/ accessed on 20 December 2021). Original structure was elucidated based on X-ray crystallography analyses at atomic resolution of 1.0A° performed by [22]. The structure can be seen in a front (left) and upright (right) overviews. Red circles show the catalytic triad consists of Ser, Asp and His, that are marked in green. (**b**) Protein alignment of cutinases isolated from fungal species with different lifestyles, including necrotrophic (e.g., *Botrytis cinerea*, *Sclerotinia sclerotiorum* and *Verticilium dahliae*), hemibiotrophic (e.g., *Fusarium solani*, *Magnaporthe grisea* and *Colletotrichum gloeosporioides*), biotrophic (e.g., *Erysiphe necator* and *Blumeria graminis*), and saprotrophic (e.g., *Apergillus flavus*, *Aspergillus nidulans* and *Aspergillus oryzae*). The conserved amino acids of Ser, Asp and His that build the catalytic triad are highlighted in red rectangles. Protein sequence alignment was performed using the tools embedded in the ClustalW2 software (https://www.ebi.ac.uk/Tools/msa/clustalw2/ accessed on 12 February 2022).

Cutinases [EC 3.1.1.74] are serine esterases belonging to the α/β hydrolase family that can break down cutin polyesters by hydrolysing the ester linkages between monomers [9,10]. The active site of cutinases consists of a catalytic triad of Ser, Asp and His, a sequence order that also corresponds to other types of lipase enzymes (Figure 1A) [11]. This triad is conserved among cutinases and can be found in the protein sequences of cutinases belonging to multiple fungal species with different lifestyles, including necrotrophic (e.g., *Botrytis cinerea*, *Sclerotinia sclerotiorum* and *Verticilium dahliae*), hemibiotrophic (e.g., *Fusarium solani*, *Magnaporthe grisea* and *Colletotrichum gloeosporioides*), biotrophic (e.g., *Erysiphe necator* and *Blumeria graminis*), and saprotrophic (e.g., *Apergillus flavus*, *Aspergillus nidulans* and *Aspergillus oryzae*) (Figure 1B). Despite these sequence similarities and conserved motifs, cutinases from fungi with different lifestyles exhibit dissimilar structural and biochemical attributes, including molecular weight, optimum temperature, optimal pH, isoelectric point, kinetic constants, substrate specificity, and thermostability (*for more information* see [12]). Pioneering work performed in the early 1960s and 1970s on fungal cutinases [13,14,15,16] paved the way for the characterisation of functional cutinases from diverse fungal species with different lifestyles. It has now been established that the progression of above-ground fungal infections relies closely on cutinase activity, which often correlates with phytopathogenicity in plant-associated pathogenic fungal species and is typically not detected in fungi that are not associated with plant hosts [17]. Recent reviews have covered the interactions between plant cuticles and pathogens [8,18,19,20,21] with a focus on the mechanisms via which cuticle-derived components act as chemical signalling molecules to elicit host plant defence responses and how cuticle imperfections affect the nature of plant-pathogen interactions. However, the role of cutinases as the driving force that helps pathogens to breach this barrier have often been overlooked, despite its key role during pathogenic attack.

In this review, we provide a synopsis of the multifaceted roles of fungal cutinases during the main four stages of infection: (i) *spore landing and adhesion to the host plant cuticle*; (ii) *spore germination on the host plant cuticle*; (iii) *spore germ tube elongation and the formation of penetrating structures*; and (iv) *penetration of the host plant cuticle and inner tissue colonisation*. We then shift our focus to the concept whether the lifestyle of a given fungi is associated with cutinase activity using previous evidence from the literature and a comprehensive molecular phylogeny tree that we have constructed herein.

## 2. Fungal Cutinases Play Multifaceted Roles during Different Stages of Infection

### 2.1. Spore Landing and Adhesion to the Host Plant Cuticle

**Figure 2:**
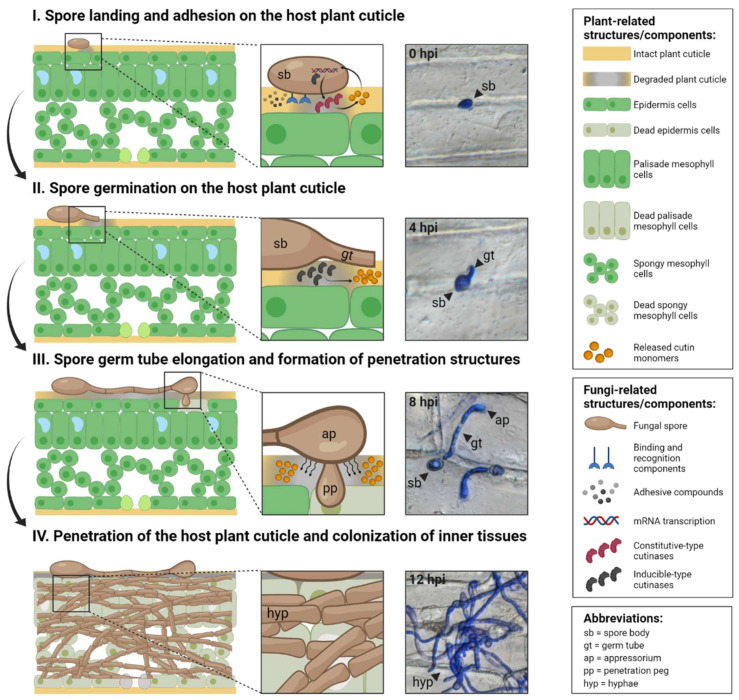
Role of fungal cutinases in the four fundamental stages of infection. Left panels: key processes in each stage. Middle panels: enlarged regions of interaction between the host plant cuticle and fungi. Right panels: micrographs of the necrotrophic fungus *Botrytis cinerea* infecting onion (*Allium cepa*) peel tissues at each stage. Right boxes: explanations of plant- and fungi-related structures/components and abbreviations. Stage (**I**) spores land and adhere to the host plant surface by secreting adhesive compounds and through dedicated binding and recognition components. ‘Constitutive-type’ cutinases actively release small amounts of cutin monomers from the host plant cuticle in a spatially-localised manner. Stage (**II**) spores hydrate by absorbing water and nutrients from the plant surface and enter a polar growth stage that leads to the formation of elongated germ tubes on the cuticle surface in preparation for penetration. Previously released cutin monomers are recognised by the spore surface, leading to the production of ‘inducible-type’ cutinases. Stage (**III**) germ tubes continue outgrowth. In many pathogenic fungi, the germ tube tip forms a swollen structure (appressorium) that facilitates cuticle penetration by applying mechanical force to the surface and piercing the cuticle via a penetration peg. Stage (**IV**) elongated germ tubes produce extended cylindrical cells (hyphae) that are typically long, filamentous and serve as the main mode of vegetative growth and/or an invasive organ that expedites cuticle penetration. The roles of fungal cutinases in cuticle penetration at these stages are demonstrated in various fungi species with different lifestyles. Figure created using BioRender (https://biorender.com/ accessed on 20 December 2021).

Spores are microscopic dispersal units produced by fungi through sexual or asexual reproduction that allow them to survive harsh conditions and reproduce. Fungal spores can be carried to the surface of the host plant by wind, water, or animal vectors. During the initial stages of infection, spores sense that they have landed on the host plant surface and remain dormant until they sense suitable conditions for infection [23] (Figure 2). These conditions vary and depend on multiple factors, including nutrient and water availability and the surface biochemistry of the host plant [24]. This initial passive, non-metabolic spore adhesion to the surface of the cuticle then allows active spore adhesion during the advanced stages of infection [25]. Fungal spore adhesion to the host plant surface involves dedicated binding and recognition components along with the secretion of adhesive compounds, such as insoluble proteins, lipids and polysaccharides [26]. These compounds, which already exist in dormant spores, are rapidly secreted towards the surface upon engagement (Figure 2) [27]. Therefore, successful and complete spore adhesion is crucial for later stages of infection and cuticle penetration, and significantly affects overall disease development [28].

The dormant spores of pathogenic fungi contain ‘constitutive-type’ cutinases, also termed previously as “sensing” cutinases, release small amounts of cutin monomers from the host plant cuticle in a spatially-localised manner (Figure 2) [29]. The molecular basis of this type of interaction has been studied in the promoter of the *Fusarium solani* f.sp. *pisi* cutinase gene using in vitro and in vivo methodologies. These efforts have identified novel regulatory elements and transcription factors that are involved in cutinase induction, including a silencing sequence that keeps basal gene expression low and affects cutinase gene inducibility; a G-rich positive-acting Sp1-like element that restores high expression levels by antagonising the silencer; a GC-rich palindrome that is vital for cutinase induction by cutin monomers; and two cutinase transcription factors (CTF1 and CTF2) that act as transcriptional inducers by binding to palindromic regions in the cutinase promoter [30,31,32,33,34,35,36]. Constitutive-type cutinase activity has been detected during early stages of infection in the dormant spores of fungal species with different infection strategies such as *Botrytis cinerea*, *Fusarium graminearium, Curvularia lunata*, *Pyrenopeziza brassicae*, *Magnaporthe grisea* and *Colletotrichum* spp. [36,37,38,39,40,41,42,43,44]. In the broad bean rust-causing biotrophic fungus *Uromyces viciae-fabae*, cutinases and other serine esterases were detected on the surface of adhesion pads formed by its spores on the bean leaves. The capacity of these spores to adhere to the host plant surface was significantly reduced when these enzymes were rinsed from the pad surface or chemically inhibited, but restored by exogenous cutinase application [45]. Thus, constitutive-type cutinases play key roles by altering the host plant surface to allow successful spore adhesion and by releasing cutin monomers from the cuticle layer that are essential for subsequent stages of infection.

### 2.2. Spore Germination on the Host Plant Cuticle

Prolonged adhesion promotes tighter and steadier attachment between the plant cuticle and spores as they secrete a polysaccharide-based extracellular mucilaginous matrix towards the plant surface. During this process, spores absorb water and nutrients from the plant surface, resulting in rapid swelling and increased volume as they become hydrated (Figure 2) [46]. Omics-based studies have indicated that the secreted extracellular matrix contains a mixture of hydrolytic enzymes, including pectinases, cellulases, and cutinases. Indeed, cutinases have been detected in the adherent spores of many fungal species, such as *Uromyces viciae-fabae* [45], *Erysiphe graminis* f. sp. *hordei* [47], *Colletotrichum gramminicola* [48], *Botrytis cinerea* [49,50], and *Fusarium solani* [29].

During this infection stage, the cutin monomers released earlier by constitutive-type cutinases are recognised by the spore surface, resulting in the protracted production of ‘inducible-type’ cutinases within minutes, which is the core driving force of cuticle penetration during later stages of infection (Figure 2) [51,52,53,54]. We have recently reviewed the signalling roles played by the released cutin monomers as well as epicuticular waxes deposited on top of the cuticle surface, during the progression of infection [18]. Consistently, various in vitro studies have shown that cutinases from different pathogenic fungi respond positively to the exogenous application of naturally extracted or chemically synthesised cutin monomers to their enzymatic buffers [16,54,55,56]. For instance, the expression of *Fusarium solani* cutinases was induced by supplementation with cutin *n*-aliphatic C_16_ and C_18_ primary alcohols [30,51], while cutinases from *Botrytis cinerea, Monilinia fructicola, Pyrenopeziza brassicae*, *Phytopthera* spp., *Ascochyta rabiei*, *Sclerotinia sclerotiorum* and *Alternaria brassicicola* were induced by the predominant cutin monomer, 16-hydroxyhexadecanoic acid [40,50,57,58,59,60]. Following complete adhesion, spores enter a polar growth phase that leads to the formation of elongated tube-like structures, known as spore germ tubes, accompanied by fundamental morpho- and cytokinetic cellular processes [46,61]. At this stage, the germ tubes lie on the surface of the cuticle in preparation for penetration. Hence, an increased production of inducible-type cutinases at this stage of infection represents the elementary phase of cuticle degradation that would be accelerated at later stages of infection.

### 2.3. Spore Germ Tube Elongation and the Formation of Penetrating Structures

As infection progresses, spore germ tubes continue their outgrowth and, in many pathogenic fungal species, their tips transform into a swollen structure known as the appressorium. This structure facilitates cuticle penetration at later stages of infection by applying a mechanical force towards the surface and piercing the cuticle using a penetration peg [26] (Figure 2). These two structures are considered to be the most specialised penetrating structures that breach the cuticle barrier, allowing the fungus to reach the inner tissue of the host plant.

Studies have shown that the cutin monomers released by both types of cutinases during earlier stages of infection promote germ tube assembly and appressoria differentiation in *Erysiphe graminis* f. sp *hordei* and *Blumeria graminis* [52,62] and *Magnaporthe grisea* [53,63]. In the latter species, upregulated expression of the cutinase gene, *MgCUT2*, was detected during appressorium maturation, while lower cutinase activity was measured in a *cut2* mutant strain that failed to form a structurally-active appressorium [42]. Similarly, appressorium disruption was detected in *Venturia ineaequalis* when cutinases were chemically-inhibited [64]. Together, these findings suggest that cutinases operate in a localised manner in germ tubes and appressoria, emphasising their central roles prior to the final penetration of the host plant cuticle barrier and the colonisation of inner tissues.

### 2.4. Penetration of the Host Plant Cuticle and Inner Tissue Colonisation

Throughout most of their life cycle, the majority of pathogenic fungi exist as extended cylindrical cells that arise from elongated spore germ tubes, known as hyphae (Figure 2). These cells are typically long and filamentous, and are the main mode of fungal vegetative growth; however, in some species hyphae act as an invasive organ that accelerates the penetration of the host plant cuticle. The role of fungal cutinases in host plant cuticle penetration has been inferred from studies showing that cutinase activity is highest in the tips of germ tubes and appressoria penetration pads, which serve as penetrating structures [65]. These crucial effects of cutinases during the final stage of infection have been demonstrated using a wide range of approaches, including reducing cutinase activity using synthetic chemical inhibitors, lowering its expression by mutation or genetic manipulation, and increasing its activity using ectopic integration or overexpression. For example, the chemical inhibition or mutation of cutinases reduced infection rates and cuticle penetration capacity in species belonging to the necrotrophic fungi *Venturia*, *Verticillium*, *Alternaria,* and *Curvularia* [39,62,66,67,68]; the hemibiotrophic *Fusarium*, *Pyrenopeziza*, *Magnaporthe,* and *Colletotrichum* [29,40,42,43,49,69,70,71,72,73]; and the biotrophic *Erysiphe* and *Uromyces* [45,52]. Although spores from some of these mutants were able to germinate on the cuticle surface, they were unable to penetrate the cuticle and their hyphal tips were augmented and abnormal, indicating ineffective penetration attempts. Remarkably, these mutants easily penetrated the cuticle when placed on wounded surfaces, further emphasising the importance of cutinase activity for this process. Consistently, studies have also shown that cutinase overexpression results in heightened and more efficient pathogenicity. For example, the overexpression of cutinase MfCUT1 from *Monilinia fructicola* increased brown rot lesion sizes in peaches, nectarines, and cherry flower petals, and was generally more virulent than the wild-type strain [74]. Another study examined *Mycosphaerella* spp., a fungus that is naturally unable to penetrate the cuticle of papaya fruit and instead infects through wounds. Interestingly, introducing *Fusarium* cutinase into *Mycosphaerella* yielded transformants that could successfully infect intact papaya fruit and penetrate their cuticle [75]. Overall, penetration through the host plant cuticle occurs directly or via the assistance of penetrating structures or both and is mediated by and tightly relies on cutinase activity. Following effective penetration, ramified invasive hyphae are established over the inner cells of the host plant.

## 3. The Association between Lifestyles of Fungi and Cutinase Activity

Cutinases have been identified in multiple fungal species with different lifestyles. Necrotrophic fungal species actively kill host plant cells and feed on their dead tissues. These species are mostly non-obligate, have a wide range of hosts, and are known to secrete copious amounts of cell-wall degrading enzymes as well as toxins [41,44]. Hemibiotrophic fungal species initiate biotrophic interactions with host plant cells followed by a necrotrophic interaction that kills the cells to feed on their dead tissues 40,43]. Moreover, it has been reported that necrotrophic and hemibiotrophic fungal species possess a cryptic biotrophic phase allowing them to persist in intercellular spaces without causing disease symptoms [76,77,78]. Biotrophic fungal species colonise living host plant cells without killing them and causing relatively little damage. These species can be obligate or facultative, typically have a narrow host range, and secrete limited amounts of cell-wall degrading enzymes [41,44]. Saprotrophic fungal species also do not kill the host plant as they can feed from entirely dead plant tissues [79]. Even though the latter two lifestyles share a similar pattern of not taking part in killing the plant cells during infection, the distinct phylogenetic separation between them is fairly predictable as biotrophic fungi still need to penetrate the cuticle, while saprotrophic fungi encounter a plant tissue that is already degraded and decayed, in which the cuticle does not present any sort of a barrier for penetration. The existence of cutinase encoding-genes in saprotrophic fungal genomes, on the other hand, plainly indicates that, although surviving on dead and decaying plant materials, they still require cutinase activity to some degree to further catalyse the complex cuticle existing in these tissues. Although each of the abovementioned lifestyles of fungi are considered distinct and are used to define a fungal species, there is a wide continuum between saprotrophy and parasitism, as well as between each of these lifestyles.

**Figure 3:**
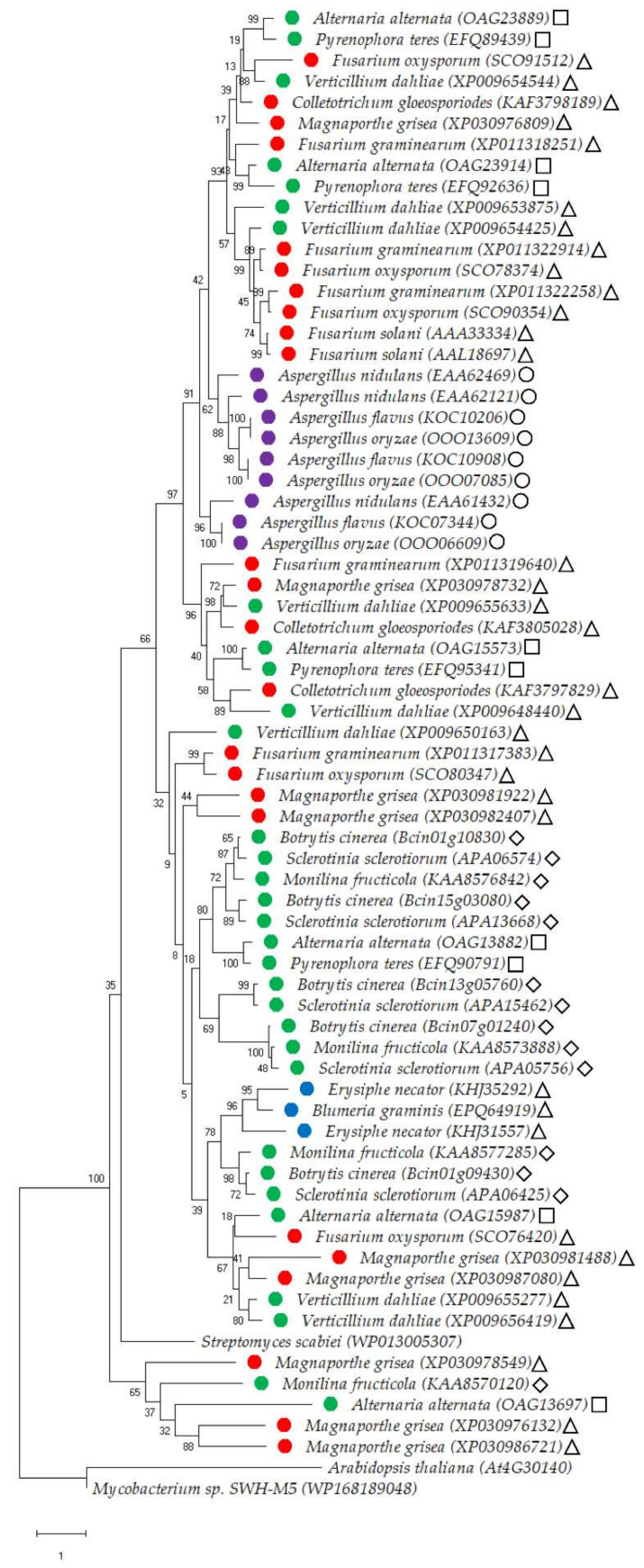
Molecular phylogenetic tree of cutinases of fungal species with different lifestyles. The phylogenetic tree was inferred by using the Maximum Likelihood method and Whelan and Goldman + Freq. model [82]. Gamma distribution was used to model evolutionary rate differences among sites [five categories (+*G*, parameter = 1.1745)]. The tree with the highest log likelihood (−26,692.25) is shown. The percentage of trees in which the associated taxa clustered together is shown next to the branches following 1000 bootstrap replications. The tree is drawn to scale, with branch lengths measured in the number of substitutions per site. There was a total of 612 positions in the final dataset. Evolutionary analyses were conducted in MEGA X [83]. The analysis involved the full protein sequences of 68 characterised and/or putative cutinases belonging to fungal species with different lifestyles based on blast analysis against the well-characterised and structurally resolved *Fusarium solani* cutinase (*AAL18697*). All of these proteins exhibited >30% sequence identity vs. the *Fusarium solani* cutinase. The isolated proteins belong to 32 necrotrophic (●), 24 hemibiotrophic (●), three biotrophic (●), and nine saprotrophic (●) fungal species. In addition, cutinases from each fungal species are classified to the following Ascomycota classes: Sordariomycetes (∆), Dothideomycetes (◻), Leotiomycetes (♢), and Eurotiomycetes (ο). For outgroups, we have used the full protein sequences of cutinases isolated from *Arabidopsis thaliana*, *Streptomyces scabiei* and *Mycobacterium* spp. Protein IDs are given in parenthesis following the species name.

Previous studies have suggested that plant-pathogenic fungi contain a conserved set of cutinases irrespective of their lifestyle [39,59,80]. In order to further investigate whether there is an association between the origin and lifestyle of plant-pathogenic fungi and cutinase type and/or activity, we generated an inclusive protein maximum-likelihood phylogenetic tree encompassing 68 functionally characterized and/or putative cutinases isolated from fungi with different lifestyles. We employed the Whelan and Goldman + Frequencies + Gamma distribution model (i.e., WAG + F + G), as suggested by a model-testing step performed prior the generation of phylogenetic tree. Finally, two bacterial cutinases and one plant-type cutinase were included in the tree as reference outgroups allowing the outcome of reliable evolutionary relationships between fungal cutinases (Figure 3).

By and large, the resulting molecular phylogenetic tree inferred relatively distinct separations of cutinases originated from saprophytic and biotrophic fungal species. For instance, all nine cutinases belonging to the saprophytic *Aspergillus* genus (comprising of *A. nidulans*, *A. flavus*, and *A. oryzae*) were grouped together forming a distinct sub-clade (Figure 3). This may suggest that fungal species belonging to this genus have similar types of cutinases that are apparently different from cutinases belonging to fungal species with different lifestyles. A similar trend was detected in the three cutinases isolated from the biotrophic fungal species of *Blumeria graminis* and *Erysiphe necator*, that formed a separate sub-clade (Figure 3). Supporting these separation patterns are the high percentages of trees in which these associated fungal species clustered together following bootstrapping (Figure 3). We fully aware that our phylogenetic tree contains a relatively low number of cutinases originating from saprophytic and biotrophic fungal species, as these were the only cutinases obtained following blast assays against the well-characterized and structurally-resolved *Fusarium solani* cutinase (>30% protein sequence identity). Alternatively, our blast assays yielded many cutinases from necrotrophic and hemibiotrophic fungal species belonging to various genera (Figure 3). Some of these were divided polyphyletically, meaning that different cutinases from the same genus/species were found in different sub-clades. Examples include nine isolated cutinases of the hemibiotrophic fungal species *Magnaporthe grisea* found in four distinct sub-clades; six cutinases of the necrotrophic fungal species *Alternaria alternata* found in four sub-clades; and eight cutinases of the necrotrophic fungal species *Verticillium dahliae* found in three sub-clades (Figure 3). On the other hand, all 10 cutinases of the necrotrophic fungal species *Botrytis cinerea* and *Sclerotinia sclerotiorum* were grouped at the same sub-clade, implying that cutinases from these two species are relatively of a similar type (Figure 3).

We also classified each of the fungal species incorporated in the molecular phylogenetic tree to different classes of the Ascomycota phylum including Sordariomycetes, Dothideomycetes, Leotiomycetes and Eurotiomycetes, all of which are evolutionarily-distinct based on their ascomatal (i.e., fruiting body) characters [81]. The saprotrophic *Aspergillus* spp. included in the phylogenetic tree all belong to the Eurotiomycetes class, and therefore may suggest that species belonging to this specific class may have a single type of a cutinase. The other three classes, Sordariomycetes, Dothideomycetes and Leotiomycetes, span throughout different clades in the phylogenetic tree, inferring that they include species with numerous phylogenetically distinct types of cutinases (Figure 3). Taken together, it appears that necrotrophic and hemibiotrophic fungal species have multiple types of cutinases, while biotrophic and saprotrophic fungal species seem to have one type of cutinase. This is an important piece of evidence that requires further attention. Phylogenetic incorporation of additional putative cutinases from fungal species with different lifestyles and Ascomycota classes will likely provide greater insight towards these remarkable concepts.

## 4. Conclusions and Future Perspective

Plant–fungi interaction is a multidimensional process governed by the secretion of various components from both the host plant and the fungus towards the cuticle surface. In particular, fungal cutinases among other enzymes are major determinants of pathogenicity and affect the capacity of the fungus to penetrate the cuticle and colonise plant inner tissues. Pathogenic fungi have different lifestyles which might influence the nature of its associated cutinases, however, this notion is poorly understood and requires further attention. As discussed above, the differences among these cutinases might be attributed to the various mechanisms and dissimilar infection strategies employed by fungi. The expression of different isoenzymes of cutinases was detected during saprophytic and parasitic stages of *Alternaria brassicicola* [84]. This suggests that different temporal infection stages of the very same fungus may also involve unalike cutinases with different characteristics that are required to execute this specific infection stage. Supporting this notion is a study that performed phylogenetic analysis and in-depth characterization of functionally and structurally diverse cutinases, implying that the genome of a single fungus might possess genes encoding different cutinase with low sequence similarities and broad functionality [85]. One study demonstrated that the secretomes (that typically include cutinases) of fungi with similar lifestyles share common attributes, but the phylogenetic history of the fungi during evolution has greater impact on the secretome compared to its lifestyle adaptation [86]. Additional bioinformatics study performed a large-scale sequence-similarity network analysis to identify common components of virulence mechanisms of major pathogenic fungi lifestyles. This approach suggested that the exploitation of cutinases is a significantly enriched function of hemibiotrophic and necrotrophic lifestyles [87].

All in all, a more comprehensive knowledge on fungal cutinases’ activity and properties is crucial not only for understanding plant-fungi pathosystems, but also due to the emerging roles of cutinases as efficient biocatalysts with manifold of applications in industrial biotechnology. These applications range from the degradation of numerous plastics synthetic polymers, enzymatic de-polymerization in treatment of post-used synthetic polymers, to bioremediation of many other substances and pollutants [12].

## Data Availability

Not applicable.
